# The Durability of Concrete Modified by Waste Limestone Powder in the Chemically Aggressive Environment

**DOI:** 10.3390/ma12101693

**Published:** 2019-05-24

**Authors:** Maja Kępniak, Piotr Woyciechowski, Paweł Łukowski, Justyna Kuziak, Rafał Kobyłka

**Affiliations:** 1 Department of Building Materials Engineering, Warsaw University of Technology, 00-637 Warsaw, Poland; p.woyciechowski@il.pw.edu.pl (P.W.); p.lukowski@il.pw.edu.pl (P.Ł.); j.kuziak@il.pw.edu.pl (J.K.); 2 Institute of Agrophysics, Polish Academy of Sciences, 20-290 Lublin, Poland; r.kobylka@ipan.lublin.pl

**Keywords:** concrete, durability, waste limestone powder, sulphate corrosion, chloride diffusion

## Abstract

The idea of sustainable development assumes that natural resources must be treated as limited goods and that waste must be managed rationally. This idea and the constant striving to reduce production costs make the use of waste materials as substitutes for traditionally used raw materials from non-renewable sources increasingly popular. In cement concrete technology, there are many possibilities to use waste as components of mortars and concretes. The subject of this paper is a fine-grained material, obtained as a by-product during the preparation of aggregate for mineral-asphalt mixtures. The aim of the research was to test the suitability of the selected type of powder, namely limestone powder, as a component of cement composites. The paper presents an evaluation of the potential of using the limestone powder as a substitute for the fine aggregate, focusing on the impact of such a modification on aspects of durability. The sulfate degradation and chloride ion diffusion in concrete were investigated. The overall desirability function has been determined. It was demonstrated that the satisfactory value of the general desirability can be attributed to most of the investigated concretes. Positive test results support the potential of replacing part of natural fine aggregate with the tested waste limestone dust without a negative impact on the durability of concrete.

## 1. Introduction

The idea of sustainable development assumes that natural resources should be treated as limited goods and the wastes should be rationally managed. Increasing amounts of collected waste, up to 2500 million tonnes per year over the world [1] encourage the search for new methods of their disposal, including recycling.

The modern engineering of building materials enables significant modification of the composites used in construction. New directions of research on the building materials include, among others, the waste management issues. The assumptions for these strategies and the constant striving to reduce production costs make the use of the waste materials as the substitutes for traditional raw materials from non-renewable sources increasingly important.

In cement concrete technology there are many possibilities of using wastes as components of mortars and concretes [2]. The waste can be used as aggregate, addition or cement component.

Production of cement (4.1 billion tons in 2017 [3] and its continuous growth is forecasted [4]) is a process absorbing large amount of mineral raw materials and the fossil fuels. At the same time, it is very energy-consuming and causes significant emission of carbon dioxide [5,6,7]. In order to reduce the negative impact of cement production on the environment, it is advisable to search for the materials that can partially replace the cement or clinker.

The demand for aggregates for the production of building materials is also constantly increasing. Restrictions resulting from the need to protect the environment reduce the possibility of using the existing natural aggregate deposits. In many countries, there is already a shortage of natural sand which can be used for the production of concrete [8,9], which in consequence leads to limitations of its extraction [10,11]. Therefore, it is necessary to search for materials that can replace or be partial substitutes for natural aggregates.

According to the EU Waste Legislation the substitution of natural components with the waste must not impair the quality of the final product. The use of waste and by-products for the production of concrete can be very wide, however, its impact on the characteristics of the final product is complex. The possibility of using waste is determined by the technology, which allows the benefits from its use to outweigh the unfavorable side effects. It is also important to eliminate the adverse impact of waste on the characteristics of the final product, i.e. concrete, to the greatest extent economically and technologically justifiable.

The current studies indicate that mineral powders have a mostly beneficial effect on the durability of cement composites. The use of natural rock powders, e.g. marble powder [12], quartz powder [13], basalt powder [14], granite powder [15], lime powder [16] has a positive effect on rheological properties, strength and durability of the cement mortars and concretes. The possibility of use of waste perlite powder as a component of cement and polymer-cement composites is also investigated [17].

Different industrial processes are the source of wastes and by-products in the form of mineral powders, and the characteristics of these wastes depend not only on the raw materials used, but also on the technological process in which they are created.

The subject of this paper is a fine-grained mineral material obtained as a by-product during production of aggregates for the hot-mix asphalt (HMA). The initial stage of HMA production is drying combined with aggregate dedusting. As a result of this process, large amount of mineral dust is accumulated in the filters of dryers and temporarily stored in silos. A small part of it is used for the production of HMA. In most cases, however, it is disposed as a filler for excavations or embankments, which definitely do not exhaust the potential of the mineral powder material. 

What is specific for HMA production is that the obtained waste includes a whole group of materials diversified due to the different raw materials, i.e., the types of rock used in production, as well as the specifics of the production process. Due to the good adhesion of the asphalt binder to the basalt and lime coarse aggregate, they are most frequently used in the production of HMA. Due to technological conditions, the time of aggregate preparation should be constant. Therefore, depending on the humidity and temperature of the prepared aggregate, it is necessary to modify the blowing force and temperature of the dryer, which results in different characteristics of the collected waste.

The aim of the article is to present an evaluation of the suitability of a selected type of waste limestone powder as a substitute for the fine aggregate in cement composites, focusing on the impact of such a modification on the properties related to durability.

The waste limestone dusts currently used in concrete technology come mainly from the production of aggregates obtained from the rock deposit. An assessment of the impact of this type of waste has been the subject of many publications, including those concerning the durability of concrete.

The permeability can be a measure of durability resulting from the resistance of concrete to penetration and movement of aggressive substances, both liquid and gaseous, inside the concrete [18]. Therefore, it is one of the key factors determining the durability of concrete. Waste limestone powder may play the role of a microfiller and thus reduce the permeability of cement slurries with their addition [19]. Different results are also presented in the publications. According to [20,21], the increase of powder content caused an increase in permeability, and thus a decrease in frost resistance of concrete.

Another factor related to the durability of concrete is its absorbability. The addition of limestone dust as a partial substitute for sand in the amount of 15% results in a decrease in concrete absorbability [22,23]. According to the authors [22,23], it is related both to a decrease in the volume of pores and a break in their continuity. The effectiveness of reducing water absorption by replacing part of cement with limestone dust depends on the particle size of the powder. The use of limestone powder with grains larger than cement grain size increases the absorbability [24,25,26].

One of the most aggressive factors causing corrosion of steel in reinforced concrete are chlorides. Uysal et al. [27] demonstrated that the addition of limestone powder, the grain size of which was greater than that of cement, increased the permeability of concrete to chloride ions. In the case of limestone dust of grain size finer than cement, this dependence is reversed-there is a reduction in the diffusion coefficient of chloride ions with an increase in the content of limestone powder in concrete [28,29,30].

Sulphates are another aggressive factor related to cement concrete. Uysal and Sumer [31], in order to evaluate the resistance to sulphate corrosion, analyzed the decrement in the strength of concrete stored in solutions: 10% magnesium sulphate and 10% sodium sulphate in relation to the strength of samples stored in water. The presented studies show that the replacement of a part of cement with limestone powder increases the resistance to sulphate corrosion. The use of limestone powder as a partial substitute for sand also increases the resistance to sulphates [32].

Studies on the application of calcium waste dust from the preparation of aggregate in concrete in HMA plants have not been published so far, except for works by Esquinas et al. [33,34]. The analysis of the influence of dolomite waste powder from HMA plants carried out by [33,34] concerned the mechanical properties and durability features of self-compacting concrete in which a traditional filler was replaced by waste. According to [34], it is possible to obtain comparable results of technological features related to self-compaction of concrete with traditional filler and waste. Concretes made of waste showed lower compression strength, lower splitting strength, lower bending strength [34]. According to [33], concrete with the addition of waste powder had a higher porosity than concrete with traditional filler. This also resulted in a higher absorbability of concrete with waste. However, both the obtained results of water absorption and the depth of water penetration from the pressure indicate that the standard conditions of an aggressive environment for both types of concrete are met. The results [33] also indicate a similar course of chloride ion penetration, a larger range of carbonization and a smaller range of sulphate ion penetration in the case of replacing a traditional filler with waste. 

## 2. Materials and Methods

The purpose of the conducted research was to determine the impact of partial substitution of the fine aggregate by the waste limestone powder on the chemical resistance of cement concrete. Concrete samples were prepared according to the experimental design. The prepared samples were subjected to the following tests: compressive strength after 28 days, resistance to chloride ions penetration and resistance to sulfate degradation.

### 2.1. Characteristic of Waste Limestone Powder

**Figure 1:**
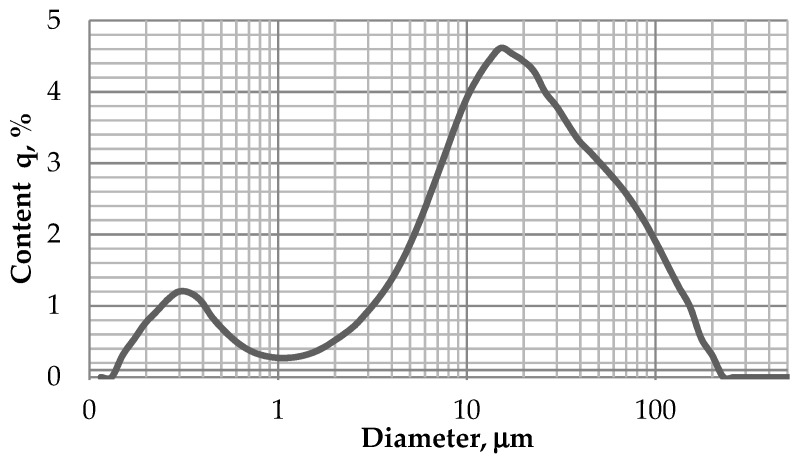
The grain size distribution curves of waste limestone powder-relative frequency plot.

The waste limestone powder used in the experiment was obtained from HMA production. The base aggregate used to that production was limestone. The basic properties of the powder are presented in the Table 1. The specific area and grain size distribution of the powder were tested using Laser Diffraction Particle Size Analyzer, Horriba LA-300 (Horiba Instruments Inc., Irvine, CA, USA). The Laser Scattering Method (LSM) was used. The sodium polimetaphosphate (NaPO_3_)_n_ (ADEVIQ – S.P.I.N. s-ka z o.o., Wrocław, Poland) was used as dispersing agent. The concentration of the solution was 0.2%. The ultrasounds were used for 1 minute to break up the potential agglomerates of particles. The obtained specific area for tested powder was 26,444 cm^2^/cm^3^. The average diameter was 27.5 μm and the mode diameter was 14.2 μm. The particle size distribution is presented in the Figure 1.

**Figure 2:**
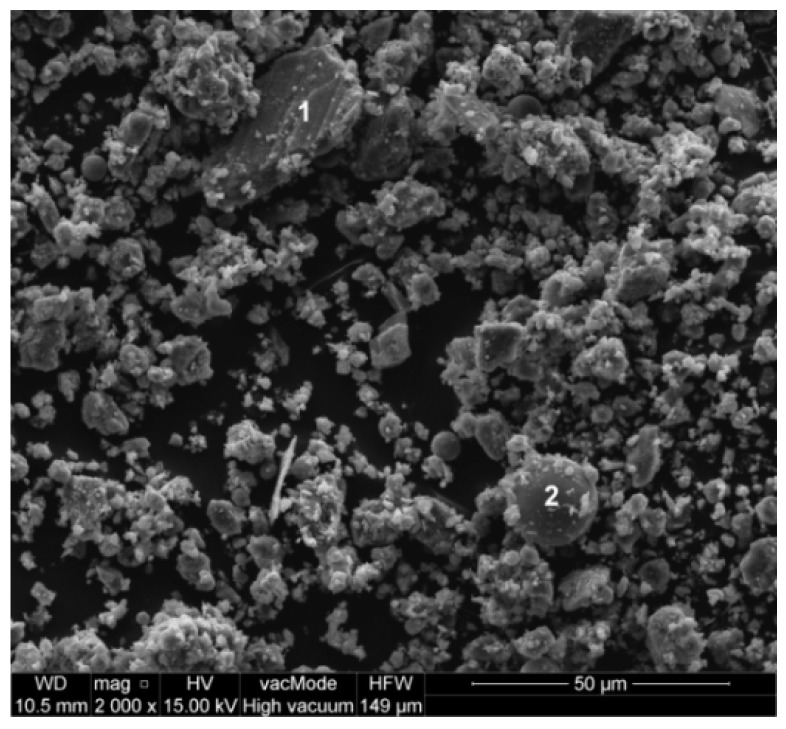
SEM image of waste limestone powder: 1—limestone grain, 2—aluminum silicate grain [35].

The chemical composition tests shows that the main component of the powder is CaO, which content calculated as CaCO_3_ is 84.85 %wt. Content of SiO_2_ is 7.06 %wt. and Al_2_O_3_ 2.38 %wt. (their carriers are aluminosilicates occurring in the form of feldspars and clay minerals, and quartz). The scanning electron microscope FEI Quanta 250 FEG (MDS, Hillsboro, OR, USA) was used for current investigation (Figure 2, [35]). The smooth, uniform shape of grains is advantageous due to the lower water demand, and consequently, good workability of the concrete mix.

### 2.2. Experimental Design

**Figure 3:**
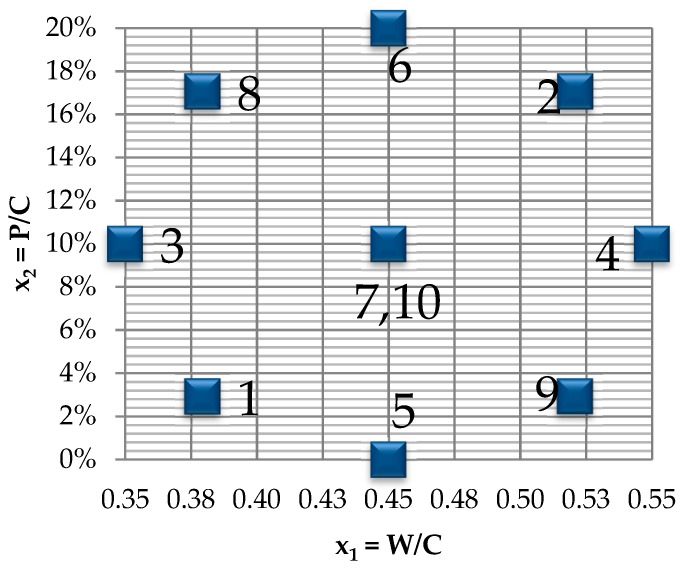
The input variables in the experimental design.

The experimental design included, as the material variables, the water/cement coefficient (W/C) in the range of 0.35 ÷ 0.55 and the level of sand substitution with the waste powder, expressed as a mass ratio of waste to cement in the range of 0–20% (P/C) which means 0–10.8% of natural sand mass. Due to the planned statistical analysis, a bi-factorial polyseck-rotal-quasiuniformal plan was adopted with a two-fold repetition of the measurement at the central point (Table 2, Figure 3) [36].

The composition of the reference, unmodified concrete mix (composition No. 5 in the experimental design) was adopted in accordance with EN 1766:2001. Therefore, the concrete mixes were prepared with the use of a fixed amount of cement CEM I 42.5 R NA HSR amounting to 375 kg/m^3^. The crushed granite aggregate of the group of fractions 2/16 and river sand were used. The grain size curve was selected according to the standard composition by EN 1766:2001 (0/2–37%, 2/8–38%, 8/16–25%). An air-entraining admixture in a constant amount of 0.2% of cement mass was applied, as well as a water reducing admixture in a variable amount dosed to obtain a constant consistency of S3 for all mixes.

### 2.3. Resistance to Chloride Ions Penetration

**Figure 4:**
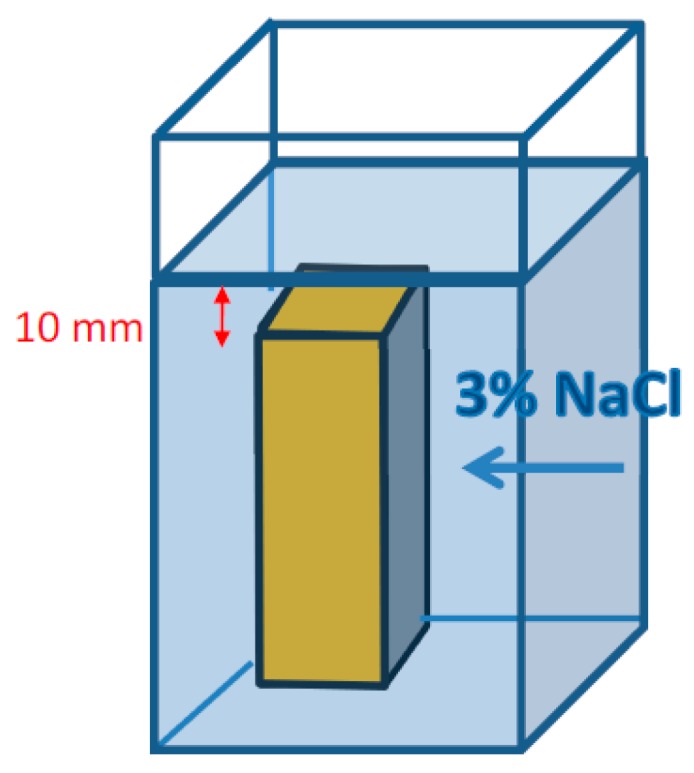
A scheme of placing the specimens in 3% NaCl solution. Yellow surfaces are coated with epoxy resin.

The transport of chloride ions in the cement concrete is a complex phenomenon. It is not just a diffusion process. Some chlorides are bound in the hardened cement paste and because the pores in the concrete are uneven in position and shape, the transport of chloride ions is not straightforward. For these reasons, the measure of chloride ion penetration in concrete is the effective diffusion coefficient (*D*_eff_). It was determined according to EN 13396, using the Fick’s law. Cubic samples of 150 mm side lengths were prepared for testing. The specimens were cured for the first 24 h covered with the plastic sheet and then were stored for 27 days in a climatic chamber (temperature t = 20 ± 2 °C; relative humidity RH ≥ 95%). After this time, the specimens were cut in half to open the structure and then protected on each side, with the exception to the cut surface, with an epoxy resin to prevent chloride ions penetration through surfaces other than the tested one. The prepared specimens were placed in 3% NaCl solution made with demineralized water. The samples were positioned so that the test surface was perpendicular to the solution level and the samples were covered with at least 10 mm of solution (Figure 4).

After 29 to 36 days the specimens were removed from the solution and the Profile Grinder Kit [37] was used to take the powdered concrete samples from different depths (up to 8–10 mm) for the determination of the chloride ions. The concrete layers were 1 mm thick. The determination of chloride ions was carried out using the Volhard argentometric method [38]. The effective coefficient of chloride ions diffusion was determined by adjusting the following equation:

(1) $$c=c_{0}\left[1-\mathrm{erf}\left(\frac{x}{2\sqrt{D_{\mathrm{eff}}t}}\right)\right]$$

where:

*c*—concentration of chloride ions in concrete at the depth x,
*c*_0_—concentration of chloride ions in the surface layer of concrete,
erf—error function,
*x*—distance from concrete surface,
*D*_eff_—effective coefficient of chloride ions diffusion,
*t*—time of penetration of chlorides into concrete.

### 2.4. Resistance to Sulfate Degradation

The sulfate resistance of cement composites is usually tested as an expansion of beam mortar specimens during exposure on sulfate solution [39,40]. Preliminary sulfate degradation tests carried out for mortars modified with waste limestone powder did not show any significant impact of the waste on the increase of length of specimens, normally observed as a result of sulfate degradation. The aim of the research was to test the resistance of concrete, in which the coarse aggregate presence would limit the measurable value of expansion. Therefore, an alternative method of testing the effect of modification of concrete with waste mineral powder on the resistance to corrosive effects of sulfates was proposed in this study. The following parameters measured in the corrosion tests were selected: compressive strength change (Δ*f*_c_), mass change (Δ*m*) and appearance of the samples-changes occurring on the surface of the specimens. In order to intensify the corrosive action of sulfates, small cubic samples with a side of 50 mm, formed by cutting the cubic samples with a side of 100 mm, were used. The specimens were cut after 28 days of curing (one day under the plastic sheet and 27 days in water under laboratory conditions). From each composition 24 samples were prepared. A summary of the number of samples to be tested is presented in the Table 3.

**Figure 5:**
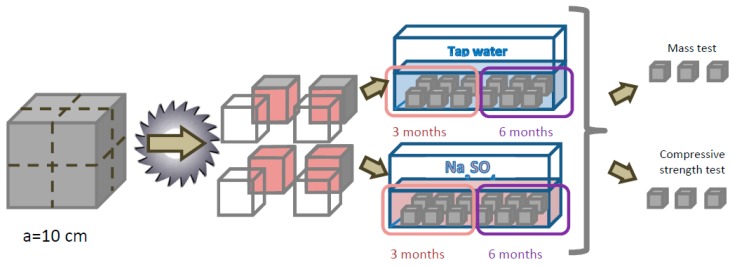
A scheme of sulfate degradation testing procedure.

The specimens to be tested for mass changes were dried to the constant mass in the oven at 105 °C and then weighed and placed in water or in the Na_2_SO_4_ solution, as appropriate. Twelve specimens from each composition were stored in water and twelve in Na_2_SO_4_ solution prepared with demineralized water. The concentration of SO_4_^2−^ ions was 10 g/dm^3^. Both the solution and the water were replaced every two weeks. After three months, the first group of samples was examined. Second group of samples was tested after six months. The specimens were tested according to the Table 3 and the external surfaces of the specimens were evaluated before the strength and mass changes measurements. In order to determine the mass changes, the specimens were dried again to the constant mass at 105 °C (Figure 5).

## 3. Results and Discussion

Conducted investigation covered the testing of the compressive strength, resistance to chloride ions penetration and resistance to sulfate degradation. The results are presented in the Table 4. 

The statistical analysis was carried out in two steps: the prediction of dependent variables: *f*_c_, *D*_eff_ and ∆*f*_c,6m_ (compressive strength change after 6 months of storing in sulfate ions solution) and finding the overall desirability function. For the prognostic calculations, StatSoft’s Statistica software was used. The generalized additive models (GAM) [41] were applied. In the statistical analysis, the partial autocorrelation function and autocorrelation function of the residual number were used [42]. The mean absolute percentage error (MAPE) was calculated. The Shapiro-Wilk’s normality test [43] was performed on the analyzed data. According to the summary statistics the coefficient W was determined for every variable and compared with the critical value *W*_critical_ = 0.842, for the number of observations *N* = 10 and the level of confidence *α* = 0.05. Variables: ∆*f*_c,6m_ (*W* = 0.918 >*W*_critical_) and f_c_ (*W* = 0.928 > *W*_critical_) had a normal distribution. The variable *D*_eff_ (*W* = 0.560 < *W*_critical_) did not have the normal distribution. Therefore, the GAM method was applied.

### 3.1. Compressive Strength

**Figure 6:**
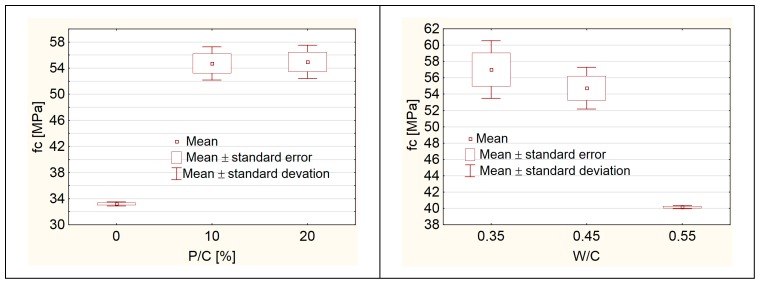
The compressive strength of concrete. Chart for compositions with constant W/C = 0.45—**left** side, chart for compositions with constant substitution level P/C = 10%—**right** side.

The compressive strength of the tested specimens increased with a W/C ratio decrease and with increase of the level of substitution of sand with the waste limestone powder (P/C) (Figure 6). This change indicates to the sealing of the microstructure of the hardened cement paste by waste limestone powder. Particularly significant increase of the compressive strength was observed up to the substitution level of 10%. After exceeding this value, the increase of compressive strength was lower.

The GAM analysis was performed for the compressive strength testing results. The following equation was found:

(2) $$f_{c}=e^{4.56937-1.88459\cdot \left(W/C\right)+1.63847\cdot \left(P/C\right)}$$

**Figure 7:**
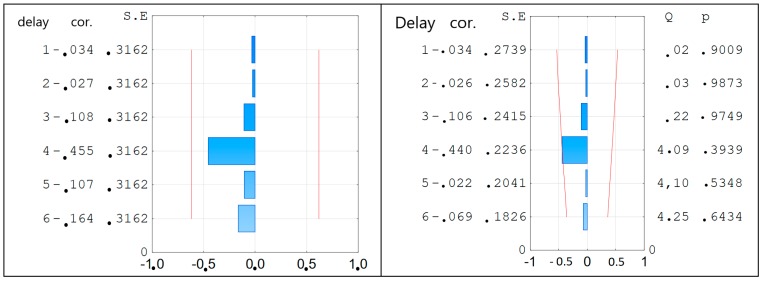
The partial autocorrelation function—**left** side and autocorrelation function—**right** side of the residual number of f_c_ equation for compressive strength.

For the obtained equation, the coefficient of determination was calculated R^2^ = 0.99 and MAPE = 1.55%. The partial autocorrelation function and autocorrelation function of the residual number were verified [35] (Figure 7). It could be concluded that Equation (2) is a regression equation and could be used to the further analysis.

**Figure 8:**
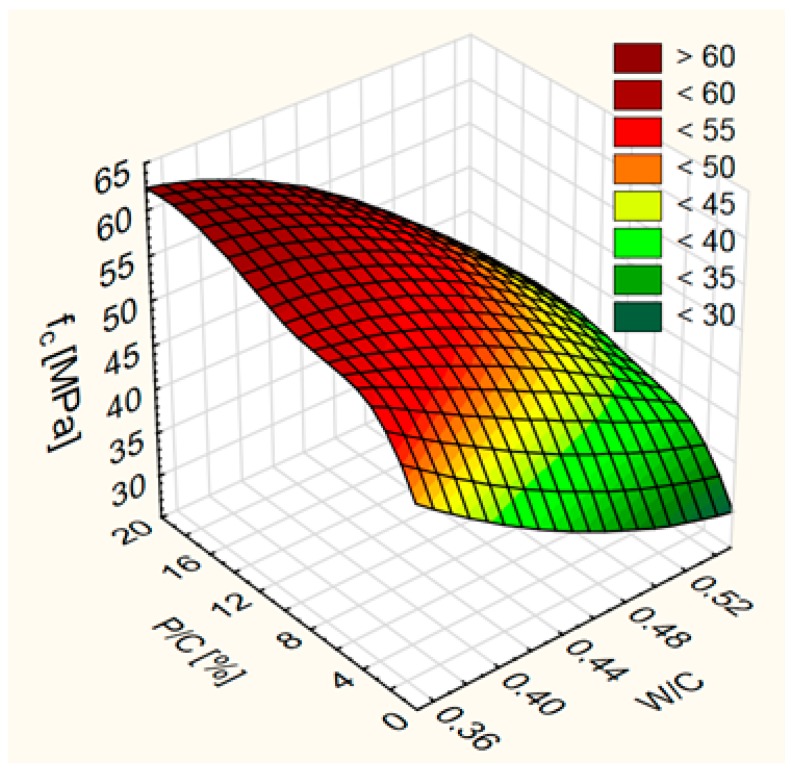
Compressive strength as a function fo water/cement ratio (W/C) and level of sand substitution (P/C).

In the tested range of W/C and P/C values, the effect of W/C on the compressive strength of concrete after 28 days of hardening is greater than the impact of P/C (Figure 8).

### 3.2. Resistance to Chloride Ions Penetration

**Figure 9:**
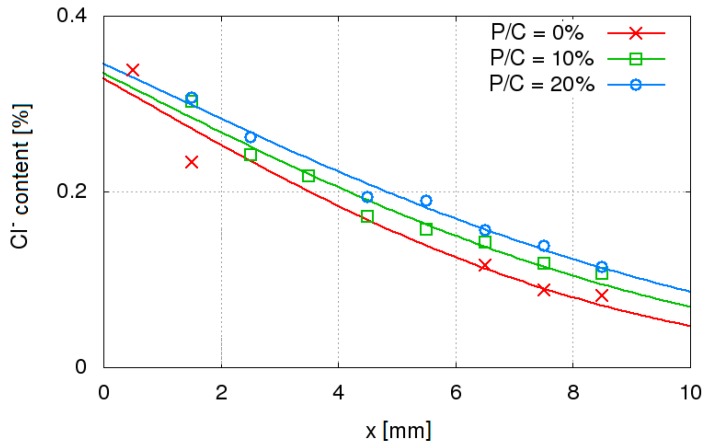
Dependence of chlorides content in concrete with W/C = 0.45 on the concrete depth (points) fitted to Equation (1) (lines).

**Figure 10:**
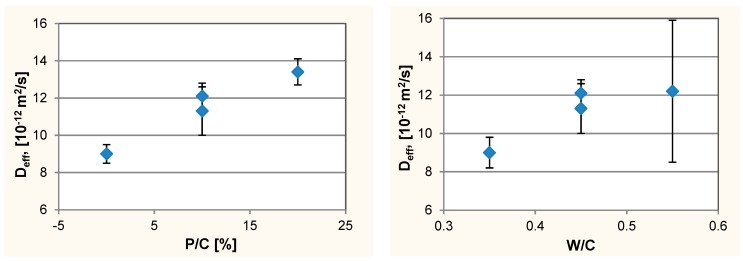
The effective diffusion coefficient of chloride ions. Chart for compositions with constant W/C = 0.45—**left** side, chart for compositions with constant substitution level P/C = 10%—**right** side.

Comparing the chloride ions concentration distributions in concretes with W/C = 0.45, an increase in the concentration of chloride ions with the increase in P/C was observed (Figure 9). This indicates the faster penetration of chloride ions into the concrete with a higher content of the waste limestone powder. The determined *D*_eff_ values confirms the faster penetration of chloride ions into the concrete modified by the limestone powder (Figure 10, Table 4). Considering the sealing of concrete and increasing f_c_ by using waste limestone powder, it was expected that the tested addition would rather result in slower diffusion of chloride ions into the concrete. Unexpected increase of *D*_eff_ with P/C increase may indicate that despite reduction of the total porosity, the limestone powder causes an increase in the capillary pores content, which determines the rate of diffusion of ions in concrete. A similar relationship, i.e., an increase in the diffusion rate despite concrete sealing, has been observed in the diffusion studies of nitrite ions [44] and chloride ions [45] in noncarbonated and carbonated concrete. In the case of diffusion of nitrite ions in concrete, it was also shown that changes in the composition of the pore liquid in concrete may cause a shift of equilibrium between the free and bound nitrite ions [46,47]. Similar relationships can be expected for chloride ions, which in the concrete form similar salts (3CaO·Al_2_O_3_·CaCl_2_·10H_2_O) as nitrite ions (3CaO·Al_2_O_3_·Ca(NO_2_)_2_·10H_2_O) [48]. These phenomena may be responsible for the higher values of the chloride ions diffusion coefficient in concretes with the addition of the waste limestone powder.

As expected, an increase in W/C resulted in an increase in *D*_eff_, i.e., an increase in the rate of chloride ions penetration into the concrete as a result of increased porosity of the concrete. Only the result for the composition No. 8 differs from this trend. However, the *D*_eff_ value for this concrete is very high and has a very big measuring error. Therefore, in the further analysis of the results, *D*_eff_ for the composition No. 8 was not taken into account.

The GAM analysis was performed for the results obtained for effective coefficient of chloride ions diffusion. Due to the high strength of the composition No. 8 specimens, there were problems with effective grinding of the sample during the test. The results obtained differed significantly from the others and were rejected.

**Figure 11:**
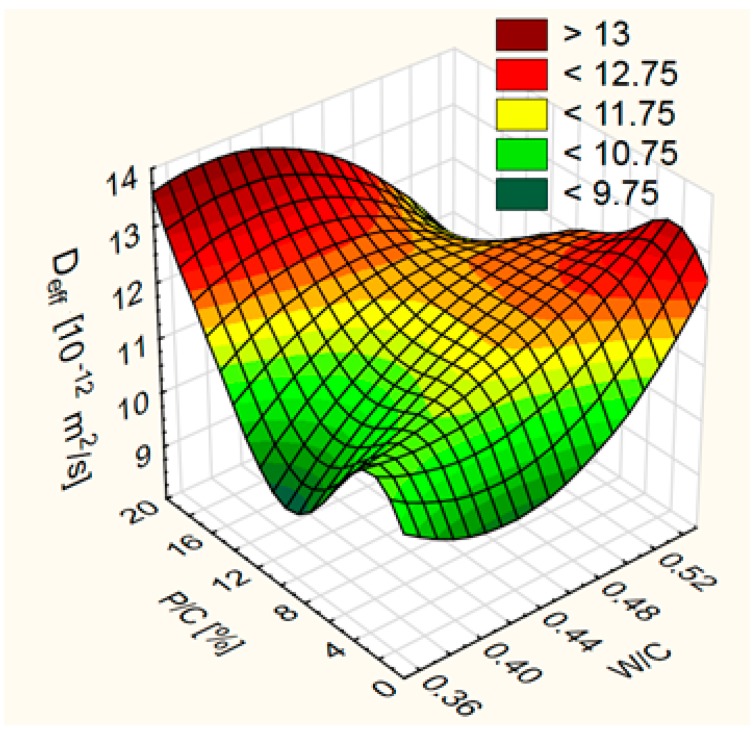
Effective diffusion coefficient of chloride ions—*D*_eff_ as a function of water/cement ratio (W/C) and level of sand substitution (P/C).

The dependence graph of *D*_eff_ as a function of water/cement ratio (W/C) and level of sand substitution (P/C) has a very unusual course in the entire range of experiment (Figure 11). Noteworthy low values of *D*_eff_ were obtained for concrete with W/C = 0.45 without limestone powder (composition No. 5) and for concrete with W/C = 0.35 and P/C = 10% (composition No. 3). Concrete mixes No. 3 and 5 were characterized by high air conten-4.0 and 6.5%, respectively. This indicates a significant role of air-entraining of the concrete in the diffusion of chloride ions and the need for further research in this field, as this topic is poorly recognized in the literature. In the work [49], the use of air-entraining admixture more often caused the *D*_eff_ increase, but in 1/3 of the tested concrete a reduction of *D*_eff_ was observed. The reasons for these observations were not offered. In [50], it was found that the addition of an air-entraining admixture causes better performance against chloride ingress. The influence of water reducing admixture on the penetration of chlorides into concrete was also observed in the work [50]. 

### 3.3. Resistance to Sulfate Degradation 

**Figure 12:**
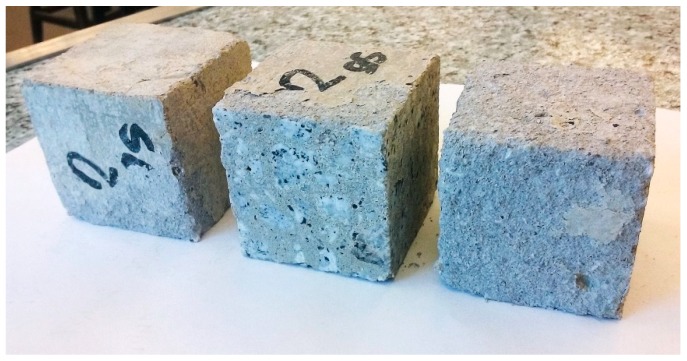
Appearance of the specimens after exposure to sulfate solution—large peeling of the surface visible (composition No. 2, W/C = 0.52, P/C = 17.07%).

**Figure 13:**
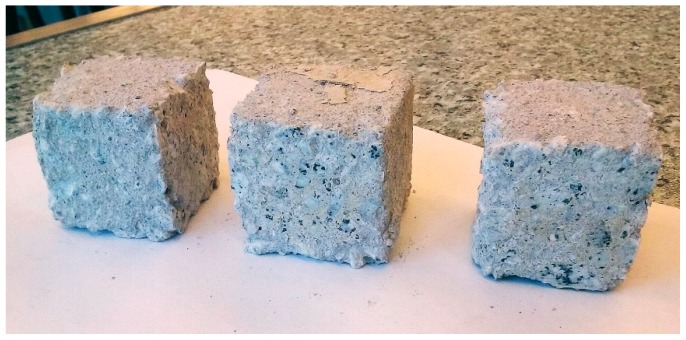
Appearance of the specimens after exposure to sulfate solution—without significant surface damage (composition No.9, W/C = 0.52, P/C = 2.93%).

After exposition of the specimens to the sulfate solution, the surfaces of the cubes were examined. Some of the specimens showed no significant surface damage, but in some cases a scaling of the surface was observed (Figure 12 and Figure 13). 

Slight changes of sample mass (from –0.6% to 0.8%) were observed after 3-months exposure to the sulfate solution. In various durability studies, 5% [51] is often taken as the critical value of weight loss. For compositions No. 7 (W/C = 0.45, P/C = 10%) and 8 (W/C = 0.38, P/C = 17.02%), no mass changes were noted. For the majority of concretes, a slight increase in the mass of the samples resulting from the formation of the corrosion products (gypsum and ettringite) in the pores of the concrete was observed. In the case of two compositions: No. 4 (W/C = 0.35, P/C = 10%) and 10 (W/C = 0.45, P/C = 10%) a small mass loss was measured due to the peeling and falling of pieces of the specimens. Composition No. 7, for which the mass change was 0% and No. 10, for which the mass change was −0.3%, were the same. This discrepancy of the results for the samples with the same composition may indicate that three months of exposure to the sulfate solution for the composition with W/C = 0.45 and P/C = 10% is a limit moment in which the samples start to peel. Samples mass changes after three months in sulfate sulution were small and do not allow to evaluate the corrosion process due to sulfate ions for the tested concretes. Mass changes did not correlate with the compressive strength changes after three months of exposure to sulfate solution. The concretes with high W/C and concretes with low W/C and P/C showed an increase in compressive strength, which means that only the pores started to be filled with corrosion products and the initial sealing of the concrete structure took place. The statistical errors in the obtained Δf_c,3m_ results are large, which may indicate that 3 months of the test is too short time to assess the effects of concrete corrosion due to sulfate ions.

**Figure 14:**
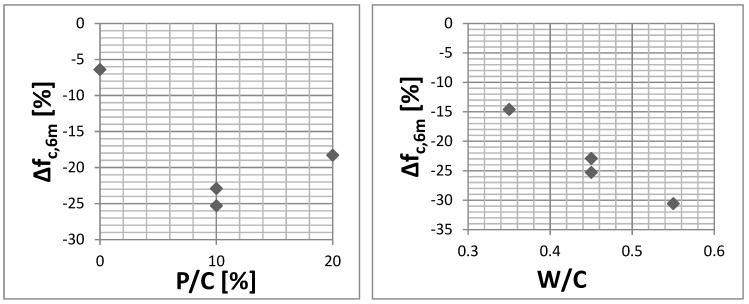
The compressive strength change after 6 months of exposition to the sulfate solution. Chart for compositions with constant W/C = 0.45—**left** side, chart for compositions with constant substitution level 10%—**right** side.

After 6 months of concrete exposure to sulfates, it was observed that the resistance to sulfate degradation decreases with increasing W/C ratio (Figure 14, Table 4). With the same W/C of 0.45, the biggest decrease in compressive strength after exposure to sulfate solution occurred at the 10% level of sand substitution by the waste. A small compressive strength loss for concrete with W/C = 0.45 without waste limestone powder (composition No. 5) can be associated with the high air-entraining of the concrete mix.

Full sulfate degradation started to develop only after six months. For this reason, the results obtained after 6 months of exposure of the concrete to the solution were further analysed using GAM method. The following equation was found (3):

(3) $$\Delta fc,6m=\{\begin{array}{c} -8.55+43.95\cdot \frac{W}{C}-1.66\cdot \frac{P}{C}-55.77\cdot {\left(\frac{W}{C}\right)}^{2}-2.86\cdot \frac{P}{C}\cdot \frac{W}{C}+11.73\cdot {\left(\frac{P}{C}\right)}^{2}\;\;for\;\frac{W}{C}<0.45\\ 6.18-23.56\cdot \frac{W}{C}-9.23\cdot \frac{P}{C}+21.52\cdot {\left(\frac{W}{C}\right)}^{2}+13.99\cdot \frac{P}{C}\cdot \frac{W}{C}+11.73\cdot {\left(\frac{P}{C}\right)}^{2}\;\;for\frac{W}{C}\geq 0.45 \end{array}$$

**Figure 15:**
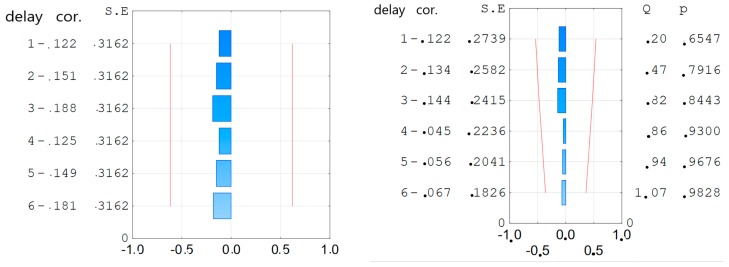
The partial autocorrelation function (**left side**) and autocorrelation function (**right** side) of the residual number of equations of resistance to sulfate corrosion.

For the obtained Equation (3) the coefficient of determination was calculated R^2^ = 0.99 and MAPE = 1.00%. The partial autocorrelation function and autocorrelation function of the residual number were verified [Cobb] (Figure 15). It could be concluded that Equation (3) is a regression equation and could be used for further analysis.

**Figure 16:**
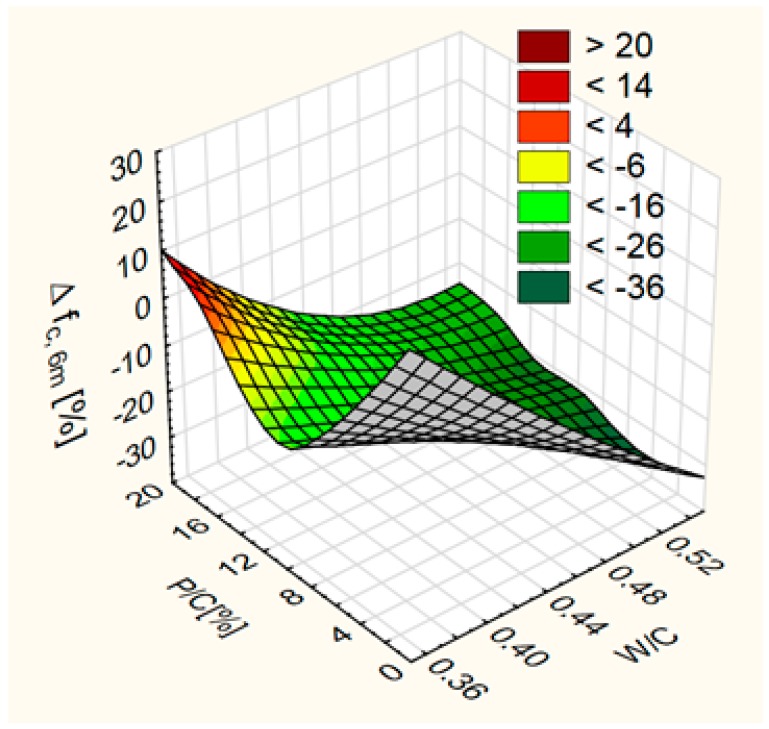
Differences of compressive strenhth after exposure to sulfate—∆*f*_c,6m_ as a function of water/cement ratio (W/C) and level of sand substitution (P/C).

The effect of W/C on the Δ*f*_c,6m_ is greater than the effect of P/C in the range of studied values (Figure 16). The biggest decreases in f_c_ were obtained for concrete with high W/C. At high W/C, increase of the limestone powder content increases the resistance to sulfate degradation. At medium and small W/C, increase of the waste limestone powder content resulted in the decrease of the sulfate degradation resistance first and then in increase.

**Figure 17:**
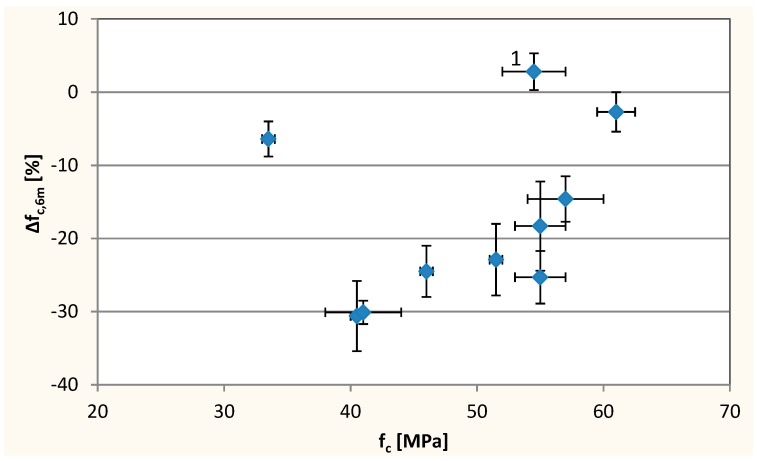
Changes of compressive strength after exposure to sulfate—∆*f*_c,6m_ as a function of compressive strength (f_c_).

The trend of the dependence of the compressive strength change of concrete after 6 months immersion in a sulfate solution on concrete strength after 28 days was observed, except to two air-entrained concretes (compositions No. 1 and 5) (Figure 17). As expected, higher Δ*f*_c,6m_ was obtained with f_c_ increase, which means greater corrosion resistance of higher strength concretes.

**Figure 18:**
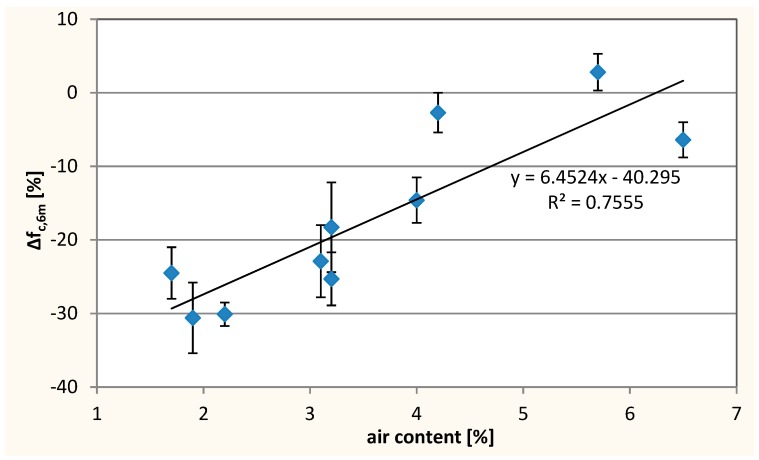
Changes of compressive strength after exposure to sulfate—∆*f*_c,6m_ as a function of air content in concrete mix.

In addition, the dependence of air-entraining of the concrete mix on Δ*f*_c,6m_ (Figure 18) was observed, indicating an increase in the resistance of concrete to sulfates with the increase of air-entraining of the concrete mix. The air bubbles could break the capillary pores continuity and cause less capillary absorption, which inhibits the penetration of sulfates ions into the concrete. In addition, sulfate salts (gypsum, ettringite) crystallizing with increasing volume could ’squeeze’ into the empty bubbles, which prevented the concrete from cracking.

## 4. Conclusions

The analysis of conducted test results showed the suitability of waste limestone powder as a partial substitute for aggregates in concrete. The maximum level of sand substitution with waste up to 20% of cement weight did not cause any significant changes in the properties related to the durability of concrete.

On the basis of the conducted research it was found that sand substitution with waste limestone powder results in:

- An increase in compressive strength of concrete. A greater increase in compressive strength was observed up to the substitution level of 10%. After exceeding this value, the increase in compressive strength was lower;
- An increase of diffusion rate of chloride ions in concrete with W/C = 0.45 with increase of waste limestone powder. This trend has not been observed in the entire tested W/C range probably due to the application of air-entering admixture and various air content in various concrete mixes. Air-entraining admixture affects the diffusion rate of Cl^–^ in concrete. Using of the air-entering admixture can reduce the penetration of chlorides into the concrete. The influence of air content in concrete on the diffusion of chloride ions is poorly recognized and should be further investigated;
- An increase of resistance to sulfate attack in concrete with high W/C (W/C = 0.55) with the increase in substitution level. In case of concrete with lower W/C, increase of content of waste limestone powder results in decrease in resistance to sulfates at low substitution level and an increase in resistance to sulfate at high substitution level

Both the phenomenon of chlorine contamination of concrete and the phenomenon of sulphate destruction are associated with the diffusion of ions in porous liquid in capillaries. The results of studies on the influence of P/C growth (i.e. increase in the share of limestone powder in concrete) on these two features are different. *D*_eff_ measurement is an issue related only to diffusion. It can be assumed that the reduction of capillary dimension in the range greater than the dimension of Cl^−^ ions favours capillary diffusion. In the case of sulphate degradation, the effects of filling pores with corrosion products are the subject of measurement. Smaller capillaries fill faster and prevent the further migration of SO_4_^2−^ ions from the solution, which inhibits the degradation process. This effect is observed only at low W/C ratios, in which the initial capillary size is small. The mechanisms described above explain different conclusions concerning the effect of calcium waste powder on chloride diffusion and sulphate degradation.

## Figures and Tables

**Table 1:** The physical properties of waste limestone powder.

Property	Value
Colour	light grey–dark grey
pH (of water slurry)	12.5
Specific density, g/cm^3^	2.65 ÷ 3.00
Bulk density (in loose state), g/cm^3^	0.7÷1.1
Chlorides content, %	0.004 ÷ 0.034

**Table 2:** Coded variables and actual experimental design; W/C (x_1_)—water to cement ratio, P/C (x_2_) —level of fine aggregate substitution with waste limestone powder, C—cement content, W—water content, P—waste limestone powder content, 0/2—sand content, 2/8 and 8/16 coarse aggregate content.

Composition No.	Coded Variables		Actual Variables		Concrete Mix Compositions [kg/m^3^]					
	x_1_	x_2_	W/C [kg/kg]	P/C [%]	C	W	P	0/2	2/8	8/16
1	−1	−1	0.38	2.93	375	142	11	711	742	488
2	1	1	0.52	17.07	375	195	64	606	689	453
3	−1.414	0	0.35	10.00	375	131	38	696	753	495
4	1.414	0	0.55	10.00	375	206	38	622	677	446
5	0	−1.414	0.45	0.00	375	169	0	696	715	471
6	0	1.414	0.45	20.00	375	169	75	621	715	471
7	0	0	0.45	10.00	375	169	38	659	715	471
8	−1	1	0.38	17.07	375	142	64	658	742	488
9	1	−1	0.52	2.93	375	195	11	659	689	453
10	0	0	0.45	10.00	375	169	38	659	715	471

**Table 3:** Number of specimens to be tested for resistance to sulfate degradation (one composition).

Test Time	Mass Changes		Compressive Strength Changes	
	Reference Samples	Samples Stored in the Solution	Reference Samples	Samples Stored in the Solution
3 months	3	3	3	3
6 months	3	3	3	3

**Table 4:** Results of compressive strength, effective coefficient of chloride ion diffusion and resistance to sulfate degradation tests.

Composition No.	Air Content in the Concrete Mix, %	Compressive Strength, *f*_c_ [MPa]	Effective Coefficient of Cl^−^ Diffusion, *D*_eff_ 10^−12^[m^2^/s]	Resistance to Sulfate Degradation			
				Mass Change after 3 Months, ∆*m*_3m_ [%]	Compressive Strength Change after 3 Months, ∆*f*_c,3m_ [%]	Mass Change after 6 Months, ∆*m*_6m_ [%]	Compressive Strength Change after 6 Months, ∆*f*_c,6m_ [%]
1	5.7	54.5 ± 2.5	12.3 ± 2.1	0.1	11.1 ± 2.5	0.5	2.8 ± 2.5
2	1.7	46.0 ± 0.5	11.1 ± 2.8	0.8	−4.8 ± 3.7	−0.5	−24.5 ± 3.5
3	4.0	57.0 ± 3.0	9.0 ± 0.8	0.4	1.4 ± 2.6	−0.4	−14.6 ± 3.1
4	1.9	40.5 ± 0.5	12.2 ± 3.7	−0.6	−8.9 ± 1.5	4.0	−30.6 ± 4.8
5	6.5	33.5 ± 0.5	9.0 ± 0.5	0.5	4.8 ± 3.2	−0.1	−6.4 ± 2.4
6	3.2	55.0 ± 2.0	13.4 ± 0.7	0.3	−11.3 ± 2.4	−1.8	−18.3 ± 6.1
7	3.2	55.0 ± 2.0	12.1 ± 0.7	0.0	−10.1 ± 1.1	−9.0	−25.3 ± 3.6
8	4.2	61.0 ± 1.5	28.0 ± 8.5	0.0	3.4 ± 4.3	0.4	−2.7 ± 2.7
9	2.2	41.0 ± 3.0	13.4 ± 2.1	0.3	6.6 ± 0.8	−5.5	−30.1 ± 1.6
10	3.1	51.5 ± 0.5	11.3 ± 1.3	−0.3	1.2 ± 1.6	−9.0	−22.9 ± 4.9

## References

[1] Agrawal D., Hinge P., Waghe U.P., Raut S.P. (2007). Utilization of industrial waste in construction material—A revive. Int. J. Innov. Res. Sci. Eng. Technol..

[2] Saikia N., Brito J. (2009). Use of industrial waste and municipality solid waste as aggregate, filler or fiber in cement mortar and concrete. Adv. Mater. Sci. Res..

[3] Zinke R.K., Werkheiser W.H. (2018). Mineral commodity. Summaries 2018.

[4] Scrivener K.L. (2012). Issues in sustainability in cements and concrete. Am. Ceram. Soc. Bull..

[5] Malhotra V.M. (2010). Global warming, and role of supplementary cementing materials and superplasticisers in reducing greenhouse gas emission from the manufacturing of Portland cement. Int. J. Struct. Eng..

[6] Van Oss H.G., Padovania A.C. (2002). Cement Manufacture and the Environment: Part I: Chemistry and Technology. J. Ind. Ecol..

[7] Celik K., Jackson M.D., Mancio M., Meral C., Emwas A.-H., Mehta P.K., Monteiro P.J.M. (2014). High-volume natural volcanic pozzolan and limestone powder as partial replacements for Portland cement in self-compacting and sustainable concrete. Cem. Concr. Compos..

[8] Arivumangai A., Felixcala T. (2014). Strength and durability properties of granite powder concrete. J. Civ. Eng. Res..

[9] Divakar Y., Manjunath S., Aswath M.U. (2012). Experimental investigation on behavior of concrete with the use of granite fines. Int. J. Adv. Eng. Res. Stud..

[10] Almeida N., Branco F., Santos J.R. (2007). Recycling of stone slurry in industrial activities: Application to concrete mixtures. Build. Environ..

[11] Arulraj G.P., Adin A., Kannan T.S. (2013). Granite powder concrete. IRACST-Eng. Sci. Technol. Int. J. (ESTIJ).

[12] Aliabdo A.A., Abd Elmoaty A.E.M., Auda E.M. (2014). Re-use of waste marble dust in the production of cement and concrete. Constr. Build. Mater..

[13] Bonavetti V.L., Irassar E.F. (1994). The effect of stone dust content in sand. Cem. Concr. Res..

[14] Abdelaziz M.A., El-Aleem S.A., Menshawy W.M. (2014). Effect of fine materials in local quarry dusts of limestone and basalt on the properties of Portland cement pastes and mortars. Int. J. Eng. Res..

[15] Chiranjeevi Reddy K., Yaswanth Kumar Y., Poornima P. (2015). Experimental study on concrete with waste granite powder as an admixture. Int. J. Eng. Res. Appl..

[16] Topcu I.B., Ugurlu A. (2003). Effect of the use of mineral filler on the properties of concrete. Cem. Concr. Res..

[17] Łukowski P. (2016). Polymer-Cement Composites Containing Waste Perlite Powder. Materials.

[18] Neville A.M. (2012). Properties of Concrete.

[19] Dobiszewska M. (2018). Kompozyty Cementowe z Dodatkiem Pyłu Bazaltowego.

[20] Rusin Z., Śwircz P. (2013). Wpływ szczelności matrycy cementowej na mrozoodporność. Budownictwo-Technologie-Architektura.

[21] Wawrzeńczyk J., Juszczak T., Molendowska A. (2016). Determining equivalent performance for frost durability of concrete containing different amount of ground granulated blast furnace slag. Bull. Pol. Acad. Sci. Tech. Sci..

[22] Almeida N., Branco F., de Brito J., Santos J.R. (2007). High-performance concrete with recycled stone slurry. Cem. Concr. Res..

[23] Celik T., Marar K. (1996). Effects of crushed stone dust on some properties of concrete. Cem. Concr. Res..

[24] Bacarji E., Toledo Filho R.D., Koenders E.A.B., Figueiredo E.P., Lopes J.L.M.P. (2013). Sustainability perspective of marble and granite residues as concrete fillers. Constr. Build. Mater..

[25] Kanellopoulos A., Nicolaides D., Petrou M.F. (2014). Mechanical and durability properties of concretes containing recycled lime powder and recycled aggregates. Constr. Build. Mater..

[26] Ramezanianpour A.A., Ghiasvand E., Nickseresht I., Mahdikhani M., Moodi F. (2009). Influence of various amounds of limestone powder on performance of Portland limestone cement concretes. Cem. Concr. Compos..

[27] Uysal M., Yilmaz K., Ipek M. (2012). The effect of mineral admixtures on mechanical properties, chloride ion permability and impermeability of self-compacting concrete. Constr. Build. Mater..

[28] Gesoglu M., Guneyisi E., Kocabag M.E., Bayram V., Mermerdas K. (2012). Fresh and hardened characteristic of self-compacting concretes made with combined use of marble powder, limestone filler, and fly ash. Constr. Build. Mater..

[29] Heikal M., El-Didamony H., Morsy M.S. (2000). Limestone-filled pozzolanic cement. Cem. Concr. Res..

[30] Hornain H., Marchand J., Duhot V., Moranville-Regourd M. (1995). Diffusion of chloride ions in limestone filler blended cement pastes and mortars. Cem. Concr. Res..

[31] Uysal M., Sumer M. (2011). Performance of self-compacting concrete containing different mineral admixtures. Constr. Build. Mater..

[32] Binci H., Kaplan H., Yilmaz S. (2007). Influence of marble and limestone dusts as additives on some mechanical properties of concrete. Sci. Res. Essay.

[33] Esquinas A.R., Alvarez J.I., Jimenez J.R., Fernandez J.M., de Brito J. (2018). Durability of self-compacting concrete made with recovery filler from hot-mix asphalt plants. Constr. Build. Mater..

[34] Esquinas A.R., Ramos C., Jimenez J.R., Fernandez J.M., de Brito J. (2017). Mechanical behavior of self-compacting concrete made with recovery filler from hot-mix asphalt plants. Constr. Build. Mater..

[35] Kępniak M., Woyciechowski P., Franus W. (2017). Chemical and physical properties of limestone powder as a potential microfiller of polymer composites. Arch. Civ. Eng..

[36] Cobb G.W. (1998). Introduction to Design and Analysis of Experiments.

[37] http://germann.org/products-by-application/chloride-profiling/profile-grinder.

[38] Vogel A.I., Mendham J. (2000). Vogel’s Quantitative Chemical Analysis.

[39] ASTM (2018). ASTM C1012/C1012M-18b Standard Test Method for Length Change of Hydraulic-Cement Mortars Exposed to a Sulfate Solution.

[40] Irassar E.F. (2009). Sulfate attack on cementitious materials containing limestone filler—A review. Cem. Concr. Res..

[41] Hastie T.J., Tibshirani R.J. (1990). Generalised Additive Models.

[42] Shimek M. (2000). Smoothing and Regression: Approaches, Computation and Application.

[43] Shapiro S.S., Wilk M.B. (1965). An analysis of variance test for normality (complete samples). Biometrika.

[44] Królikowski A., Kuziak J. (2010). Study on the diffusion of penetrating corrosion inhibitors-Nitrite ions in concrete. Ochrona przed Korozją.

[45] Ngala V.T., Page C.L. (1997). Effects of carbonation on pore structure and diffusional properties of hydrated cement pastes. Cem. Concr. Res..

[46] Li L., Sagüés A.A., Poor N. (1999). In situ leaching investigation of pH and nitrite concentration in concrete pore solution. Cem. Concr. Res..

[47] Tritthart J., Banfill P.F.G. (2001). Nitrite binding in cement. Cem. Concr. Res..

[48] Balonis M., Glasser F.P. (2011). Calcium nitrite corrosion inhibitor in Portland cement: Influence of nitrite on chloride binding and mineralogy. J. Am. Ceram. Soc..

[49] Jóźwiak-Niedźwiedzka D. (2012). Estimation of chloride migration coefficient in air-entrained concretes containing fluidized bed combustion fly ash. Arch. Civ. Eng..

[50] Mohammed T.U., Hamada H. (2003). Durability of Concrete Made with Different Water. Reducing Chemical Admixtures Under Marine Tidal Environment. ACI Mater. J..

[51] De Almeida R. Resistance of high strength concrete to sulfate attack: Soaking and drying test. Proceedings of the International Conference “Durability of Concrete”.

